# Wetting and deposition characteristics of air-assisted spray droplet on large broad-leaved crop canopy

**DOI:** 10.3389/fpls.2023.1079703

**Published:** 2023-01-20

**Authors:** Yinlong Jiang, Zhou Yang, Xing Xu, Dongying Shen, Tingting Jiang, Bowei Xie, Jieli Duan

**Affiliations:** ^1^ College of Engineering, South China Agricultural University, Guangzhou, China; ^2^ Guangdong Laboratory for Lingnan Modern Agriculture, Guangzhou, China; ^3^ School of Mechanical Engineering, Guangdong Ocean University, Zhanjiang, China; ^4^ College of Electronic Engineering (College of Artificial Intelligence), South China Agricultural University, Guangzhou, China

**Keywords:** air-assisted spray, large broad-leaved canopy, surface wettability, spray coverage, droplet density

## Abstract

Precision and efficient pesticide spraying is an important part of precision agriculture, banana is a large broad-leaved plant, with pests and diseases, has a high demand for spraying and pest control. The purpose of this study was to clarify the wettability of different pesticides on the banana leaf surface, and the effects of nozzle type and working parameters on the deposition distribution performance under air-assisted spray conditions. The wettability test results of different pesticides on banana leaf surfaces showed that the wettability of the adaxial side was always stronger than that of the abaxial side, the smaller the surface tension of the droplets, the better the wettability on the surface. The spray experiment was carried out on the previously developed air-assisted sprayer with the latest developed intelligent variable spray control system. Three types of nozzles were used to spray with different combinations of working parameters. The deposition distribution performance on the banana leaf surface was obtained by image processing using a self-compiled program. The experimental results show that the nozzle type, wind speed, and spray pressure have significant effects on the deposition distribution performance. Through the study of the interaction and coupling effect of nozzle type and working parameters on the spray droplet deposition distribution on both sides of banana leaves, the results show that under the conditions of hollow cone nozzle, 0.5Mpa spray pressure and 3-5 m/s wind speed, the spray coverage and droplet density are in the optimal state. This is mainly due to the low spray pressure and/or wind speed is not enough to make the banana leaves vibrate and improve the performance of pesticide deposition. excessive spray pressure and/or wind speed will cause large deformation of banana leaves and make them airfoil stable, which reduces the surface deposition performance. It is of great significance for promoting sustainable and intelligent phytoprotection.

## 1 Introduction

Broad-leaved crops have the characteristics of large leaves, closed canopy, and staggered branches and leaves. Banana, as a typical large broad-leaved crop (Zhang et al., 2021; Jiang et al., 2022a), mainly grows in more than 100 countries in tropical and subtropical regions, where the temperature is hot and humid, and diseases and insect pests occur frequently (Guo et al., 2020). In the process of banana growth, spraying chemical pesticides to control diseases and insect pests and promote plant growth has become a key operation. Pesticide spraying directly affects banana yield and fruit quality, which in turn affects the economic benefits of the entire banana industry (Robson et al., 2007). Due to the lack of banana orchard pesticide spraying equipment, the over-spraying method was used to improve the deposition of pesticides, but another issue that caused public concern was the pollution of the ecological environment caused by the drift and loss of pesticides over-spraying (Barraza et al., 2020; Sinha, 2020). Therefore, improving the utilization rate of pesticides and achieving high coverage and deposition of pesticides on the target leaf surface has become a hot spot of current research, which is also an important part of precision agriculture.

Spray method, droplet size, and pesticide properties have always been the main influencing factors for improving the deposition performance of mechanized sprays (Ferguson et al., 2016). In previous studies, the air-assisted spray technology was used to fully disturb the canopy of the fruit tree to achieve air replacement and improve the deposition efficiency on both sides of the leaves (Xi et al., 2020; Gu et al., 2022; Wu et al., 2022). Use coarse nozzle to produce coarse droplets to reduce spray drift, and improve droplet size according to the operating conditions to prevent droplets from bouncing on the leaf surface (Hilz and Vermeer, 2013; Alidoost Dafsari et al., 2021). By adding surfactants, the surface tension of spray droplets can be changed, the wettability of pesticide droplets on the leaf surface can be improved, and the spray deposition performance can be improved (Nairn et al., 2014; Carvalho et al., 2017; Zhang and Xiong, 2021). However, a large number of these studies have focused on dwarf dense fruit trees and field crops, with almost zero studies on broad-leaved plants.

Air-assisted spray, on the one hand, improves the spray deposition performance inside the canopy through the principle of air replacement, and on the other hand, changes the potential energy and kinetic energy of the droplets deposited on the leaf surface through the aerodynamic response of the leaves, which affects the interaction between the droplet and the leaf surface (Xi et al., 2020; Zhang et al., 2022). Li et al. respectively studied the prediction model of the air-assisted spray droplet deposition state on citrus, litchi, longan, and guava leaves, and the results showed that appropriately increasing the wind speed to increase the aerodynamic response speed of the leaves can achieve the purpose of improving the spray coverage on the target leaves (Li et al., 2020; Li et al., 2021). Compared with other spindle canopy plants, it is an important way to solve the bottleneck of pesticide application in the banana industry to develop a banana orchard air-assisted spray technology and equipment with strong penetration and disturbance ability to ensure that pesticides can be fully deposited on both the sides.

Nozzle type and spray pressure determine droplet size, uniformity, spray flow rate and spray shape, which are important parameters affecting spray quality (Ferguson et al., 2016; Li et al., 2022b). Hołownicki et al. studied the spray performance of hollow cone nozzle TR80 and flat fan air-inclusion nozzle ID90 under three different spray pressures, and the results showed that the effect of nozzle type on droplet density was extremely significant on the adaxial side, and significant on the abaxial side of the apple leaves, meanwhile, the droplet density of fine droplet TR80 nozzle was significantly higher than that of coarse droplet ID90 nozzle (Hołownicki et al., 2021). Griesang et al. research on nine types of flat fan nozzles under two flow rates and four working pressures shows that the working pressure has a great influence on the spray volume distribution, which is the lowest CV% under the pressure of 300kpa (Griesang et al., 2022). Too small droplets may drift, and too large droplets may accumulate, slide or bounce, thus affecting the spray performance (Ding et al., 2022). Therefore, choosing the appropriate nozzle type and spray pressure can effectively improve the spray quality.

By changing the physicochemical properties of the spray droplet, surfactants can change the size of the droplets generated by the nozzle on the one hand, and improve the wettability of the spray droplets on the leaf surface on the other hand (Song et al., 2021; Li et al., 2022c; Zhao et al., 2023). However, due to the various characteristics of surfactants, the half-life of various surfactants in plant leaves and soil was not fully studied and understood, which will also bring a new problem, that is, soil environmental pollution caused by the use of a large number of surfactants (Meng et al., 2019). The study found that the improvement effect of non-ionic adjuvant Agral 90 and silicone adjuvant Silwet L-77 on the droplet size of pure water spray was diametrically opposite, while the physicochemical properties of pesticides themselves are extremely complex, and it is difficult to predict whether the effects of different surfactants interacting with different pesticides will be positive or negative (Sanderson et al., 1997; Stainier et al., 2006). Due to these uncertainties, this study will focus on the effects of nozzle type, wind speed, and spray pressure on the spray deposition performance under the condition of air-assisted spraying, without considering the related research on surfactants.

Spray coverage, droplet density, and pesticide deposition amount are important indicators for evaluating spray deposition performance (Shan et al., 2022). A large number of studies have used artificial collectors such as Mylar and water-sensitive paper (WPS) to visualize spray droplet distribution (Li et al., 2022a; Wang et al., 2022). However, due to the different wettability of natural plant leaves, artificial collectors cannot truly reflect the spray coverage and droplet density on the target leaf surface, which has also confirmed by our previous research (Xiang, 2020; Jiang et al., 2021; Jiang et al., 2022b). Therefore, in this study, banana leaves were directly used as collectors rather than any artificial collectors.

Pesticide spraying is a key task in crop protection, and nozzles are a necessary crop protection product in pesticide spraying. In the research on the efficacy of crop protection products, a large number of researchers have reported the effects of wind speed, and nozzle type on droplet size, spray drift, spray deposition, and so on. However, most studies have adopted artificial collectors, which were quite different from the actual application of pesticide spraying. At the same time, most of them did not consider the wettability of pesticides on the target leaf surfaces and the special needs of air-assisted spray in large broad-leaved plants. The purpose of this study was to determine the wetting characteristics of different pesticides on the surface of banana leaves, and to study the effects of nozzle type, spray pressure, wind speed and the interaction of these factors on the distribution of spray on natural leaves of banana plants. Analyze the spray coverage percentage and the droplet coverage density on both sides of banana leaves under different working conditions, and then guide the decision-making of the operation model of mechanized pesticide spraying in banana orchards.

## 2 Materials and methods

### 2.1 Experimental materials

The banana plants used in the experiment were cultivated from the campus of South China Agricultural University, aged 5 months. To achieve the best effect of banana disease and pest control, following the pollution-free production requirements of bananas, refer to GB/T8321 (Guideline for safety application of pesticides) and GB4285 (Standards for safety application of pesticides). Given the most disease and nutritional requirements of bananas, according to the occurrence rules of diseases and pests, three pesticides: imidacloprid (emulsifiable concentrate (EC), pest control), carbendazim (suspension concentrate (SC), disease control), and *Luyebao* (emulsion, oil in water (EW), growth promotion) were selected to carry out experimental research (Huang and Wei, 2017). According to the usage range of the selected pesticides and the instructions for the usage method, the three pesticides were prepared with 600 times dilution for reserve.

### 2.2 Pesticide wettability test

The wettability of pesticide solutions on the surface of banana leaves can directly affect the deposition state and spraying quality of pesticides. To study the wettability of different pesticide solutions on the surface of banana leaves and their influence on the deposition performance, a contact angle measuring instrument (JC2000D1, Shanghai Zhongchen Digital Technology Apparatus Co., Ltd., China) was used to test the contact angle (CA) and rolling angle (RA) of the three pesticide solutions on the banana leaf surfaces. As in our previous research (Jiang et al., 2021), the droplet size of the pesticide used in the test was 8μL, and the CA was determined by droplet shape analysis according to the ASTM D7334 standard.

### 2.3 Air-assisted sprayer and key components

#### 2.3.1 Air-assisted sprayer

To study the deposition characteristics of pesticide droplets on the surface of banana leaves, a new spray control system (Yang et al., 2021) was developed on the previously developed sprayer (Yang et al., 2019). The sprayer was composed of three parts: an air delivery module, a pesticide spraying module, and the spray control system module. The air delivery module includes fan unit, relay, and high-frequency pulse generator, and the wind speed was adjustable and can provide a stable wind field. The pesticide spraying module includes pump, spray pressure control components, the pipeline for pesticide solution, and nozzles, and the spray pressure was adjustable, nozzle can be replaced, can spray a certain amount of pesticide stably. The spray control system module was a programmable logic controller (PLC, FX3U), which is connected to the industrial touch screen (MCGS) through the RS232-485 serial communication module. It can realize command input, data refresh display and storage, and the remote signal input command can be received through the wireless remote-control receiver, the control system can adjust the spray working parameters by using a remote-control device or inputting commands on the industrial touch screen according to requirements.

#### 2.3.2 Nozzle and application parameters

Pesticides were atomized by three nozzles of three types (Guangdong Boyuan Spray Technology Co., Ltd., China), respectively: (1) Standard hollow cone nozzle AA1/8-SS-2-3W (AA2-3W), which produces a hollow circular spray performance. (2) Standard solid cone nozzle BB1/8-SS-2.8W (BB2.8W), which produces a solid circular spray performance. (3) Standard flat fan nozzle CC1/8-SS-11005 (CC11005), which produces a striped spray performance. Under the same pressure condition, the spray flow rate of the three types of nozzles was the same (the flow rate was provided by the nozzle manufacturer), to exclude the influence of spray dosage on the surface deposition characteristics of banana leaves. For each type of nozzle, three spray pressures of 0.3MPa, 0.5MPa, and 0.7MPa were used respectively.

#### 2.3.3 Fan and application parameters

Wind field was the key link of the air-assisted spray. The wind field was generated by three fans (THB2048HG) mounted on the sprayer. The command was sent by wireless remote control to control the high-frequency pulse output by the high-frequency pulse generator in the main controller module to change the fan rotation speed, to control the intensity of the wind field. A portable wind direction and speed tester were used to measure the wind speed at a spray distance of 1.0m. In this experiment, four levels of wind speed were selected: 0m/s (no wind), 3m/s, 5m/s, and 7m/s.

### 2.4 Experimental design

The experiment was carried out in November 2021 at the outdoor test site of the College of Engineering, South China Agricultural University. The air relative humidity was 65-75%, the temperature was 14-18°C, and the ambient wind speed was 0.1-0.4 m/s. The experimental variables and parameter settings of this test were shown in **Table 1**. A total of 36 times independent experiments were carried out in 3 types of nozzles, 3 types of spray pressures, and 4 types of wind speeds. The environmental conditions of each group of experiments were as same as possible, and the differences between groups were negligible.

**Table 1:** Experimental variables and parameters.

Variables	Parameters
Pesticide type	Imidacloprid, Carbendazim, *Luyebao*
Nozzle type	AA2-3W, BB2.8W, CC11005
Spray Pressure (MPa)	0.3, 0.5, 0.7
wind speed (m/s)	0, 3, 5, 7
Travel speed (m/s)	0.5

**Figure 1:**
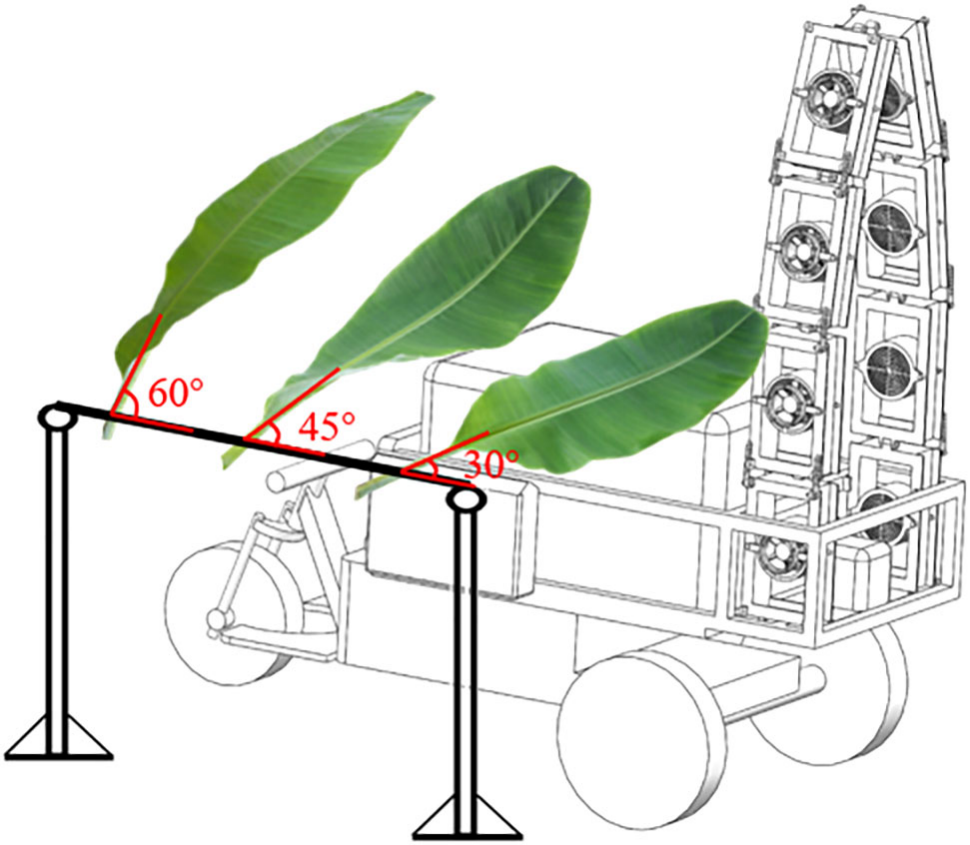
Schematic diagram of the experimental site.

The experiment site was shown in **Figure 1**. Each group of experiments consists of three banana leaves fixed on the horizontal beam through the petiole. The angles between the first to the third banana leaves and the horizontal ground were 30°, 45°, and 60°, respectively, to fully restore the change of leaf inclination angle at different growth stages. The air-assisted sprayer runs at a constant speed of 0.5 m/s from the spray distance of 1.0m. The fan will produce horizontal airflow to assist pesticide spraying and the vibration of banana leaves.

### 2.5 Statistical analysis

After the end of each group of experiments, the digital camera was used to quickly save the droplet deposition images on both side of the banana leaves, and then 2 target areas of 1.5 cm × 1 cm were intercepted as the analysis objects of droplet deposition characteristics to evaluate the spray performance. The uniformity of droplet distribution was usually used to evaluate spray deposition characteristics. The two parameters used to reflect the droplet distribution were spray droplet coverage percentage (spray coverage, *P*) and droplet coverage density (droplet density, *D*), which can be expressed as:


$$P=\frac{A_{R}}{A_{T}}\times 100\%$$

$$D=\frac{C_{N}}{S_{T}}$$

Where, *P* — the spray droplet coverage percentage, that is, the percentage of the area covered by the droplets per unit area. *A_R_* — the total number of pixels in the area covered by the droplets on the target area. *A_T_* — the total number of pixels in the target area. *D* — the droplet coverage density, that is, the number of droplets per unit area; *C_N_* — the number of droplets on the target area; *S_T_* — the area of the target area.

Matlab2019a software programming was used to implement image segmentation and droplet distribution statistics on the target area. The deposition characteristics of droplets in each group were presented by means, standard deviations, Sum of Squares, and the ratio of the sum of squares of each factor to the total sum of squares (Percent of Total) at 6 sampling points of 3 different banana leaves. The measured data of each group were processed separately, and both sides of banana leaves were analyzed independently.

In the statistical analysis of the obtained data, to clarify the influence of the three test factors on the spray deposition performance of both sides of the banana leaves. The ratio between the sum of squares of each factor and the total sum of squares (Percent of Total) was used to determine the influence degree of the independent variables. To evaluate the influence of different factors such as nozzle type, wind speed, spray pressure, and their interaction on the spray deposition performance on both sides of banana leaves. Using IBM SPSS Statistics 23 software, multi-way analysis of variance (ANOVA) was used to analyze and process the obtained data, and Duncan’s multiple range test was used to compare the mean values within a 95% confidence interval.

## 3 Results and analysis

### 3.1 Wettability of different pesticides on the banana leaf surfaces

The contact angles (CA) and rolling angles (RA) of 8μL droplets of three different pesticides on both sides of banana leaves were shown in **Table 2**. The RA of 8μL pesticide droplets was always bigger than 90°, this is mainly due to the special microstructure and chemical composition of the banana leaf surface forming a stable solid-liquid composite wetting interface, which has a certain adhesion to the pesticide droplets, showing a high adhesion. The test and analysis of the three pesticides showed that the CA was always less than 90°, the surface of the banana leaf showed a positive response to the pesticide wetting, and the CA on the adaxial side was always bigger than that on the abaxial side. This is consistent with the performance of water droplets on the banana leaf surface. The surface of the banana leaf exhibits Janus wettability, and the adaxial side wettability was always stronger than the abaxial side.

**Table 2:** Wettability of three pesticides on banana leaf surface.

	Imidacloprid	Carbendazim	*Lvyebao*
Adaxial Side	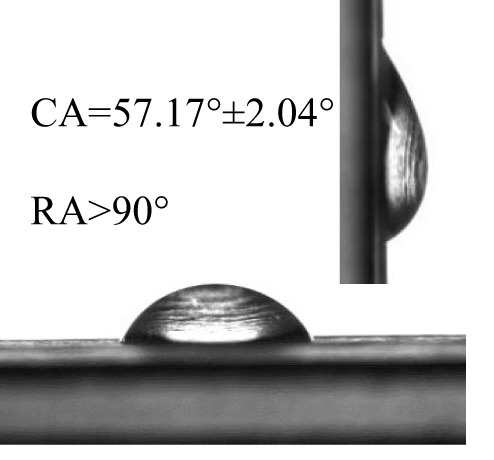	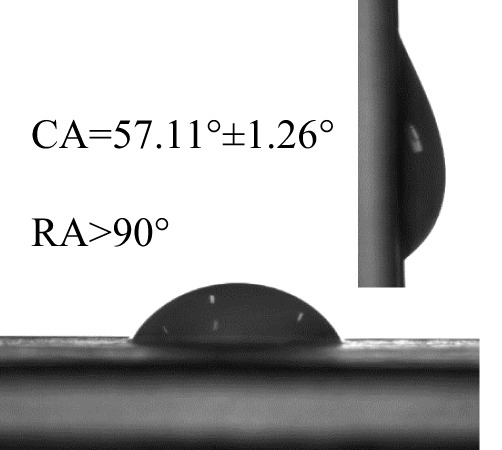	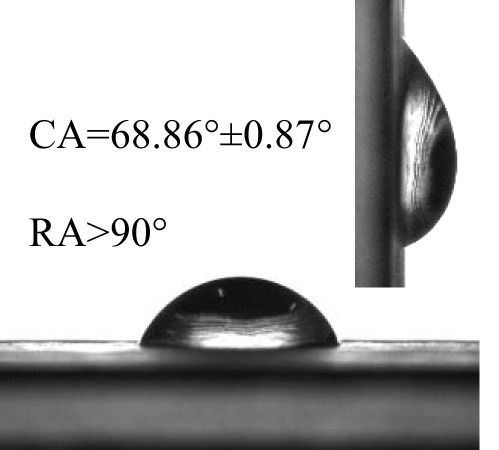
Abaxial Side	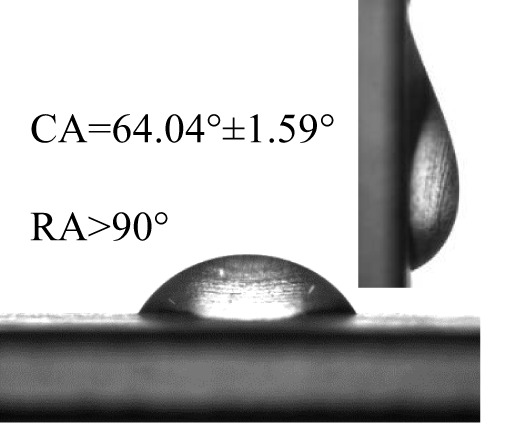	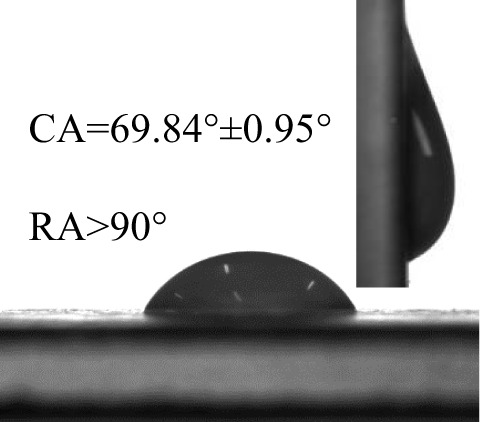	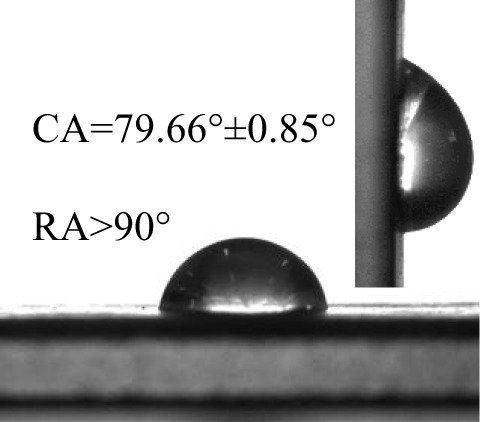

**Figure 2:**
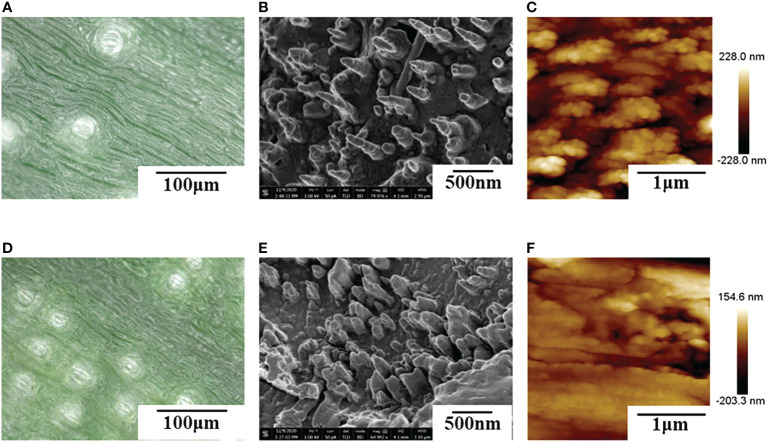
Surface microstructure of banana leaves. **(A–C)**: adaxial side, **(D–F)**: abaxial side, **(A, D)**: ultra-depth of filed 3D microscope (VHX-5000) imaging, **(B, E)**: Field emission scanning electron microscopy (Verios 460) imaging; **(C, F)**: Atomic Force microscope (Dimension edge) imaging.

The results of **Table 2** showed that imidacloprid and carbendazim had similar wettability, and the wettability was better than that of *Luyebao*, which was mainly determined by the inherent surface tension of the pesticide itself. The wettability difference between the adaxial and abaxial sides was mainly affected by the microstructure and chemical composition of the surface. As shown in **Figure 2**, the sharp and large-spacing nanopillars structure on the adaxial side and the round and small-spacing nanopillars structure on the abaxial sides make them show Janus wettability (Jiang et al., 2021; Jiang et al., 2022b). The difference in wettability between pesticides is due to the active ingredients of different solutions. On the one hand, these active ingredients improve wettability by improving surface tension. On the other hand, by softening the crystalline wax in the cuticle, the permeability and fluidity of pesticide droplets on the cuticle are enhanced, and the wetting area of pesticide droplets on the surface of banana leaves is increased (Schönherr et al., 2000). Since the main components of *Luyebao* are amino acids, the pollution to fruit quality and the environment is small. Therefore, in the study of air-assisted spray experiment, we selected carbendazim, which has slightly poorer wettability among the other two pesticides, as the research object to reveal the effects of different nozzle types and working parameters on the spray deposition performance on both sides.

### 3.2 Droplets deposition and distribution

**Figure 3:**
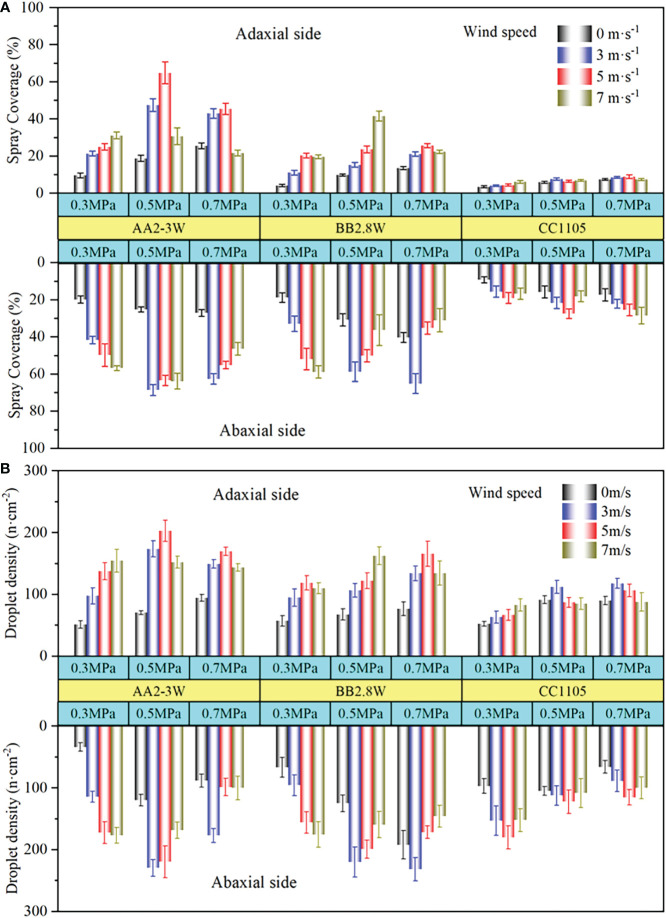
The histogram of spray coverage **(A)** and droplet density **(B)** on banana leaf surface.

The effects of three factors on the droplet deposition and distribution on both sides of the banana leaf under different levels were analysed. The experimental results confirmed that the nozzle types and working parameters have different performance on the distribution of spray deposition on the banana leaf surface. **Figure 3** is a histogram of spray coverage (A) and droplet density (B) of carbendazim air-assisted spray on the banana leaf surface.

It can be intuitively observed from **Figure 3**, whether it is spray coverage or droplet density, the spraying performance of the three nozzles were as follows: Hollow cone nozzle AA2-3W was the best, solid cone nozzle BB2.8W was second, and the flat fan nozzle CC1105 was the worst, and the spray deposition distribution on the abaxial side was always better than that on the adaxial side. Specifically, when the spray pressure of hollow cone nozzle AA2-3W was 0.3Mpa, the spray coverage and droplet density show an increasing trend with the increase of wind speed, and when the spray pressure was bigger than 0.3Mpa, the spray droplet distribution increases first and then decrease with the increase of wind speed. The solid cone nozzle BB2.8W presents a spray deposition performance similar to the hollow cone nozzle AA2-3W on the abaxial side of the banana leaf. However, on the adaxial side of the banana leaves, when the spray pressure was 0.3Mpa, the spray deposition distribution performance improved with the increase of wind speed, when the wind speed reached 7m/s, the spray deposition distribution performance decreases slightly. When the spray pressure was 0.5Mpa, the spray deposition distribution was always increasing. When the spray pressure was 0.7Mpa, the spray deposition distribution performance is the same as the abaxial side, showing a trend of increasing first and then decreasing. The flat fan nozzle CC1105 shows a spray performance similar to the hollow cone nozzle AA2-3W on the adaxial side of the banana leaf. On the abaxial side, when the spray pressure is less than 0.7Mpa, the spray coverage and the droplet density show a trend of first increasing and then decreasing. When the spray pressure reaches 0.7Mpa, the spray coverage increases with the increase of wind speed, but the droplet density showed a trend of increasing first and then decreasing.

Compared with hollow cone nozzle AA2-3W and solid cone nozzle BB2.8W, the spray deposition distribution performance of flat fan nozzle CC1105 shows a lower spray coverage but corresponding to a higher droplet density, which is particularly obvious on the adaxial side of banana leaves. In addition, with the increase of spray pressure, the spray coverage and droplet density almost all showed a trend of first increasing and then decreasing or first increasing and then remaining stable. However, the spray deposition distribution results of flat fan nozzle CC1105 on the abaxial side of banana leaves showed that the spray coverage increased with the increase of spray pressure, and the droplet density decreased with the increase of spray pressure, which was the only result that showed a significant negative correlation among all the test results. To explain this phenomenon more reasonably, we carried out an analysis of variance in the spray coverage and droplet density of carbendazim air-assisted spray on both sides of banana leaves. The analysis of variance (ANOVA) summary table was shown in **Table 3**.

**Table 3:** The ANOVA summary of spray coverage (a) and droplet density (b) on banana leaf surface.

Source of Variation	df	Adaxial Side		Abaxial Side	
		Sum of Squares	Percent of Total	Sum of Squares	Percent of Total
(a). Spray Coverage Percentage (*P*)					
Main effects:					
NT (Nozzle Type)	2	23578.52	51.18	32813.57	47.65
WS (Wind Speed)	3	5672.76	12.31	14821.65	21.52
SP (Spray Pressure)	2	3817.70	8.29	2122.18	3.08
Interactions:					
NT*WS	6	5439.96	11.81	5064.29	7.35
WS*SP	6	1656.69	3.60	5369.62	7.80
NT*SP	4	1645.55	3.57	980.57	1.42
NT* WS*SP	12	3630.77	7.88	5210.53	7.57
Error	216	629.66	1.37	2484.29	3.61
Total		46071.60		68866.69	
(b). Droplet Coverage Density (*D*)					
Main effects:					
NT (Nozzle Type)	2	76425.03	22.65	72170.36	12.79
WS (Wind Speed)	2	111895.72	33.16	125007.00	22.15
SP (Spray Pressure)	2	44183.53	13.09	33761.19	5.98
Interactions:					
NT*WS	6	45694.82	13.54	20330.31	3.60
WS*SP	6	10603.10	3.14	90669.14	16.07
NT*SP	4	4069.94	1.21	122496.94	21.71
NT* WS*SP	12	21596.98	6.40	49216.39	8.72
Error		22991.83	6.81	50622.00	8.97
Total	216	337460.96		564273.33	

The results of variance analysis showed that the effects of nozzle type, wind speed, spray pressure, and their interaction on spray coverage percentage (*P*) and droplet coverage density (*D*) were significant (sig. < 0.01). The analysis of variance showed that the strongest factor influencing the spray coverage on both sides of banana leaves was the nozzle type, accounting for 51.18% and 47.65% of the total variation, respectively. The second factor was wind speed, accounting for 12.31% and 21.52% of the total variation. Spray pressure accounted for 8.29% and 3.08% of the total variation of treatment. Although the contribution of spray pressure was lower than that of wind speed, it played an important role as wind speed, especially in the adaxial side of the banana leaves, which accounted for 12.31% and 8.29% of the total variation, respectively, with similar influence performance.

For droplet density, wind speed was the strongest influencing factor, accounting for 33.16% (adaxial side) and22.15% (abaxial side) of the total variation, followed by the nozzle type, accounting for22.65% and 12.79% of the total variation, and spray pressure accounted for 13.09% and 5.98% of the total variation respectively. Although the results of ANOVA show that the intensity of the influence of each factor on the droplet density from strong to weak are wind speed, nozzle type, and spray pressure. However, we found that the effects of nozzle type, wind speed, and spray pressure on the droplet density differed slightly from spray coverage. Besides, on the abaxial side of banana leaves, the interaction between nozzle type and spray pressure (NT*SP) and the interaction between wind speed and spray pressure (WS*SP) accounted for 21.71% and 16.07% of the total variation, which was almost opposite to the change rule of droplet coverage on the abaxial side of banana leaves. This is mainly because spray pressure is the only variable used to characterize the spray flow rate in this study, and it is coupled with the wind speed, which affects the transport performance of pesticide droplets, resulting in some extreme situations such as partial aggregation and slippage of the droplets. Therefore, more spray droplets and larger droplet size resulted in a larger spray coverage area rather than larger droplet coverage density due to droplet aggregation and other reasons under the coupling influence of wind speed, spray pressure and collision transport.

It was found that the spray deposition distribution performance on the abaxial side was much better than that on the adaxial side. The hollow cone nozzle AA2-3W has a much better spray deposition distribution performance than the solid cone nozzle BB2.8W and the flat fan nozzle CC1105. For the same nozzle, with the increase of spray pressure, the deposition distribution of air-assisted spray droplets increases first and then decreases, with the increase of wind speed, the distribution performance of spray deposition gradually increases under low spray pressures, but when the spray pressure reaches 0.5Mpa, the spray deposition distribution performance showed increasing first and then decreasing. the spray deposition distribution of the flat fan nozzle CC1105 showed a negative correlation on the abaxial side of the banana leaf.

### 3.3 Difference between the adaxial side and abaxial side

From the experimental results of the wettability of different pesticides on the surface of banana leaves, it is not difficult to find that due to its special microstructure and chemical composition, the adaxial side always shows better wettability than the abaxial side. However, air-assisted experimental results show that the spray coverage and droplet density on the abaxial side were higher than the adaxial side. We believe that this is mainly due to the fixed mode of the banana leaves on the horizontal beam (as shown in **Figure 1**) and the disturbance of the horizontal airflow during the spray process, which makes it possible that the amount of spray deposition on the abaxial side more than that on the adaxial side. Such a fixed mode makes the abaxial side of the banana leaf can directly collide with the spray droplets, while the adaxial side always requires forced disturbance of the airflow to optimize the droplet transport channel and enhance the droplet’s orbital movement ability to indirectly realize the interaction between the droplets and the leaf surface. As large broad-leaved herbaceous plants, this is also the biggest difference between banana plants and apple, litchi, and other plants.

For the 36 groups of experimental treatment results, except for the slightly different droplet density of the flat fan nozzle CC1105, the spray deposition performance on the abaxial side was always better than that on the adaxial side. This is also good news for crop protection by spraying chemical pesticides, because a large number of research results show that the abaxial side of banana leaves has become the main occurrence area of banana plant diseases and pests due to its suitable temperature, not being directly washed by rain, and not being exposed to direct sunlight (Huang and Wei, 2017). Therefore, the higher spray coverage and droplet density on the abaxial side of the banana leaves is of positive significance for crop protection by chemical pesticide spraying.

### 3.4 Interaction analysis between influencing factors

#### 3.4.1 Interaction between nozzle type and wind speed

**Figure 4:**
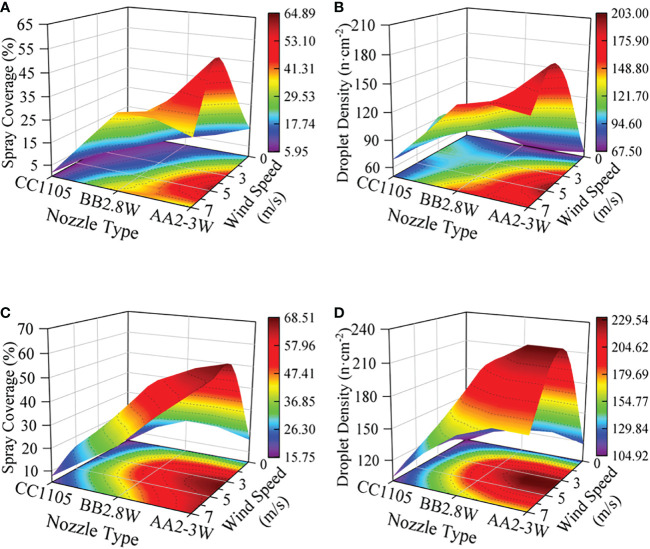
Response of Spray Deposition Distribution to Nozzle Type and Wind Speed (SP = 0.5 MPa). **(A, B)**: adaxial side, **(C, D)**: abaxial side, **(A, C)**: spray coverage, **(B, D)**: droplet density.

**Figure 5:**
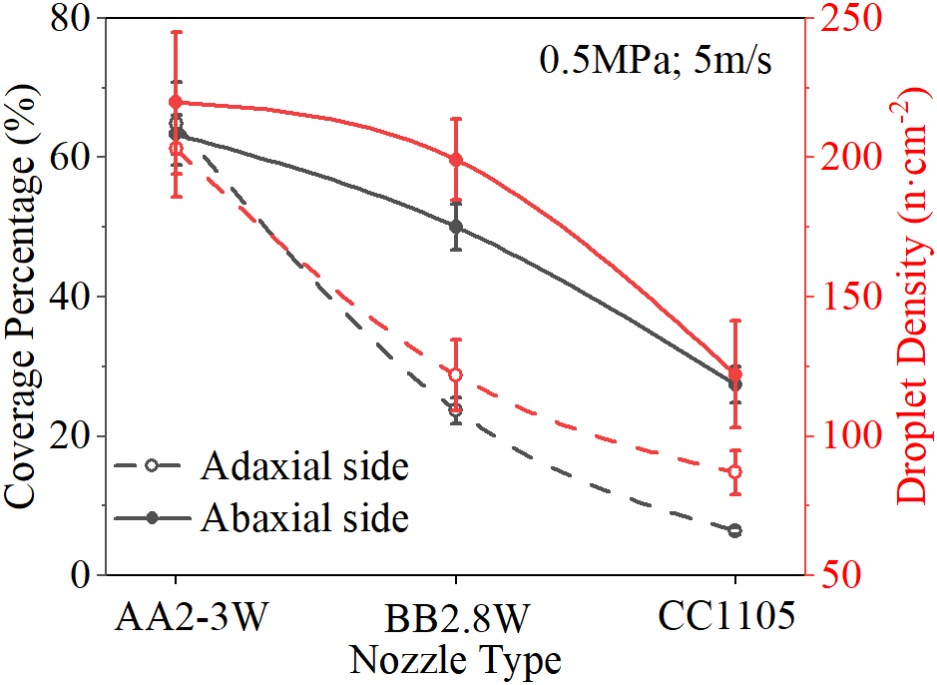
Response of Spray Deposition Distribution to Nozzle Type (SP = 0.5 MPa, WS = 5 m/s).

Different nozzle types directly determine the spray shape, droplet size, and other key factors that affect the spray deposition distribution. **Figure 4** shows the spray deposition effects of three types of nozzles under 0.5MPa spray pressure, and the results show that AA2-3W nozzles have the best deposition performance. **Figure 5** shows the spray deposition distribution of different nozzles under the conditions of 0.5MPa spray pressure and 5m/s wind speed. The results show that the influence of nozzle type on the deposition effect of the abaxial side was better than that of the adaxial side.

We think that the effect of nozzle type on spray deposition on banana leaf surface was similar to the leaf-tips preference of wheat and corn leaves for pesticide deposition, which may be caused by the elasticity of the leaves (Gart et al., 2015). the hollow cone nozzle and the solid cone nozzle have the same spray distribution width in any direction. The only difference is that solid circular distribution compared with the hollow circular ring distribution, the spray central was affected by the impact load carried by the droplet itself, which will cause stronger deformation of the banana leaf and further affect the spray deposition. However, the flat fan nozzles spray fan-shaped droplet flow, and the spray deposition shape presents a strip shape. It is not difficult to find that during the spraying process, the droplet flow and the banana leaves present different relative angles, which will directly lead to a significant difference in the spray deposition distribution performance. In addition, a smaller spray range produces a larger spray droplet size, and a more concentrated spray shape makes the droplets themselves carry a bigger impact load, the bigger impact load on the banana leaf surface makes a stronger deformation, which enhances the bounce, aggregate, and slip of spray droplets, and affects the spray deposition and distribution performance.

#### 3.4.2 Interaction between spray pressure and wind speed

**Figure 6:**
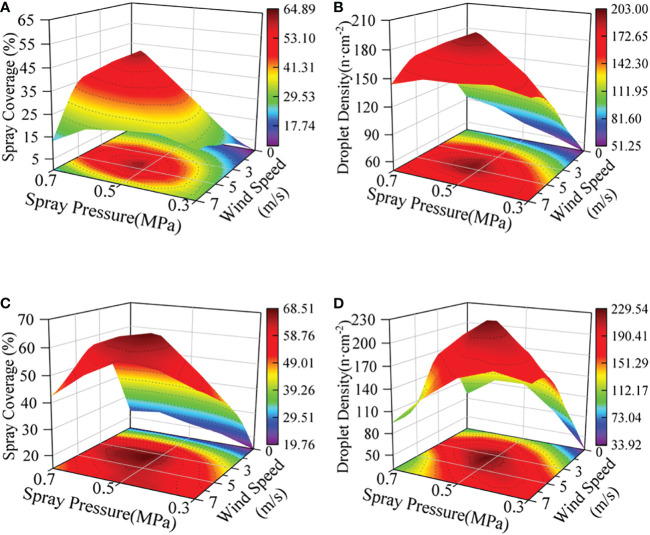
Response of Spray Deposition Distribution to Spray Pressure and Wind Speed (AA2-3W). **(A, B)**: adaxial side, **(C, D)**: abaxial side, **(A, C)**: spray coverage, **(B, D)**: droplet density.

**Figure 7:**
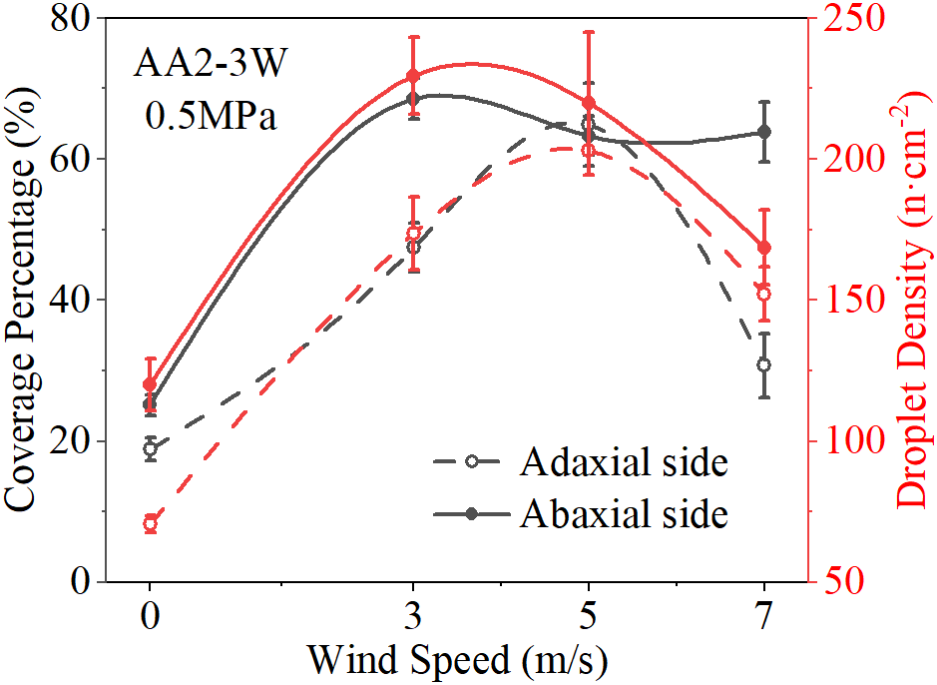
Response of Spray Deposition Distribution to Wind Speed (AA2-3W, SP = 0.5MPa).

The result of ANOVA shows that wind speed is the strongest factor affecting the spray deposition distribution without considering the nozzle type. **Figure 6** shows the spray deposition effect of the AA2-3W nozzle at different wind speeds. The results showed that with the increase of wind speed, especially with the increase of wind speed and spray pressure at the same time, under the coupling effect of wind speed and spray pressure, spray coverage and droplet density showed a trend of increasing first and then decreasing. **Figure 7** shows the response of spray deposition performance of the AA2-3W nozzle with the change of wind speed under 0.5MPa spray pressure. The results show that the spray deposition performance on the abaxial side is better than on the adaxial side, and the best spray deposition performance was achieved at the wind speed range of 3 m/s to 5 m/s.

**Figure 8:**
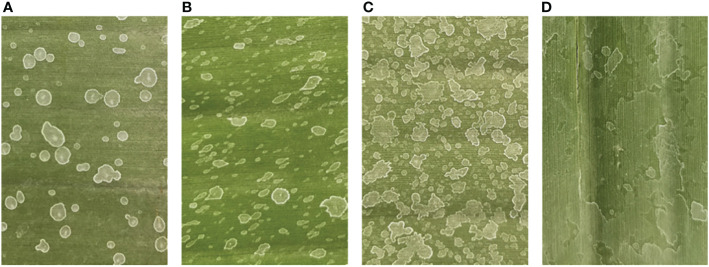
Spray droplets deposition incomplete **(A)**, uniform **(B)**, accumulate **(C)** and slippage **(D)**.

Further observation and analysis, we found that under the conditions of low wind speed and/or low spray pressure, the low spray coverage and low droplet density were mainly caused by inadequate and incomplete droplet coverage (**Figure 8A**). With the increase of wind speed and/or spray pressure, the spray performance was improved and gradually begins to show a uniform distribution state (**Figure 8B**), at which time the spray performance reaches the optimal state. When the wind speed and/or spray pressure were further increased, the droplets began to aggregate (**Figure 8C**), at this stage, the spray coverage had little influence, and the droplet density began to decrease. However, if the wind speed and/or spray pressure were further increased, the droplets will continue to aggregate and slippage, leaving the target leaf surface (**Figure 8D**), at this time, the spray coverage and droplet density were both reduced.

Besides, banana plants as large broad-leaved plants. Another problem brought about by the increase of wind speed is that the huge banana leaves will be curled and deformed after being subjected to the strong wind load and the strong impact load of droplets themselves, presenting a stable airfoil shape. At this time, even if the forced airflow optimizes the droplet transport channel and enhances the droplet transfer ability, it is difficult to produce effective spray deposition distribution on the adaxial side, this is particularly prominent in the spray experimental of flat fan nozzle CC1105. In addition, the elastic deformation of the banana leaves caused by the increase in wind speed makes the surface of the banana leaves and the direction of spray mist flow tend to be parallel, the contact area is reduced. And the wettability of the pesticide droplets on the abaxial side was worse than that on the adaxial side, it is more prone to slippage and other behaviors under the action of external force, which creates a positive condition for the slippage of the droplets on the banana leaf surface. Therefore, through our research, we believe that neither too small nor too high wind speeds can meet the pesticide spraying requirements of bananas, which are typical of large broad-leaved plants.

#### 3.4.3 Interaction between nozzle type and spray pressure

**Figure 9:**
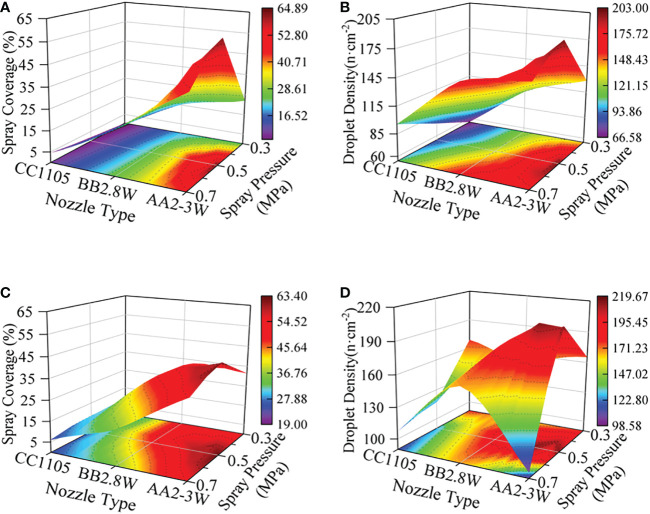
Response of Spray Deposition Distribution to Nozzle Type and Spray Pressure (WS = 5 m/s). **(A, B)**: adaxial side, **(C, D)**: abaxial side, **(A, C)**: spray coverage, **(B, D)**: droplet density.

**Figure 10:**
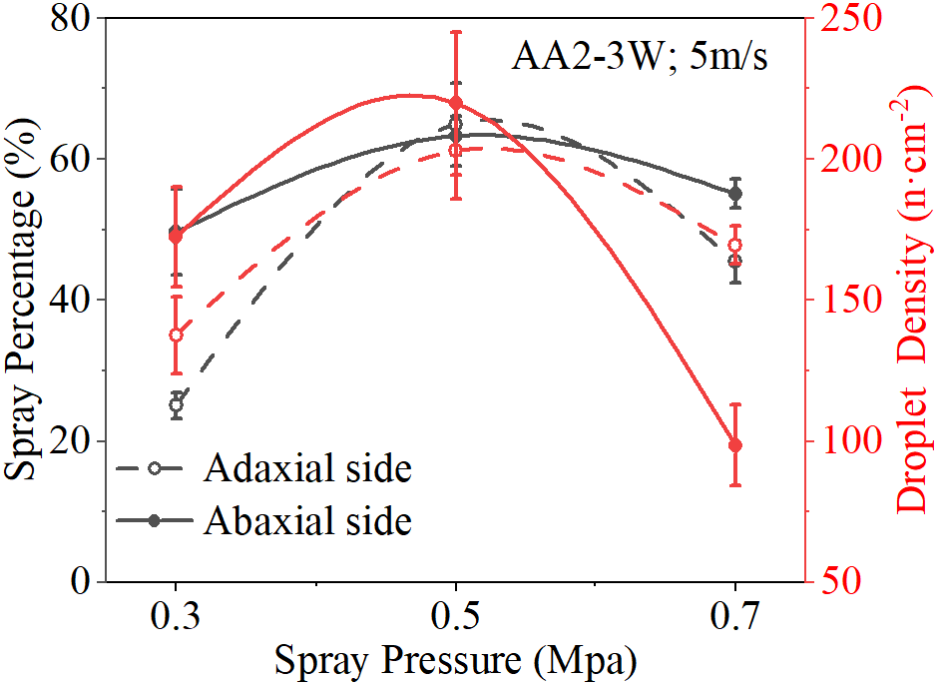
Response of Spray Deposition Distribution to Spray Pressure (AA2-3W, WS = 5 m/s).

The spray pressure is another main factor affecting the deposition and distribution of spray droplets after the nozzle types and the wind speed. Lower spray pressure produces a smaller flow rate and larger particle size droplets, which have smaller initial velocities. Higher spray pressure produces a larger flow rate and smaller particle size droplets, which have larger initial velocities. When the initial velocity of the droplets is low, if there is without the assistance of airflow, the kinetic energy of the droplets decreases rapidly and sinks under the action of gravity. Therefore, the combined effect of spray pressure and wind speed was particularly important. **Figure 9** shows the spray deposition effect of AA2-3W nozzles under different spray pressures, which is similar to the effect of wind speed on spray deposition. The result shows that spray deposition performance first increases and then decreases with the increase of spray pressure. **Figure 10** shows the spray deposition effect of the AA2-3W nozzle with the change of spray pressure under the wind speed of 5 m/s. When the spray pressure was 0.5 MPa, the spray deposition performance was the best.

According to the ANOVA results of the interaction of various factors in **Table 3**, it was found that the primary factor affecting the spray coverage and droplet density on the adaxial side of the banana leaves was nozzle type*wind speed (NT*WS), which respectively accounted for 11.81% and 13.54%. On the abaxial side of the banana leaves, the wind speed*spray pressure (WS*SP) has become the primary factor affecting the spray coverage, accounting for 7.80%, the nozzle type*spray pressure (NT*SP) has become the primary factor affecting the droplet density, and its influence degree was much higher than that of any main effects, accounting for 21.71% of the total variation, and the influence degree of wind speed*spray pressure (WS*SP) was closely followed, accounting for 16.07%. This discovery caused us to speculate on the possible relationship between the collision, rebound, wetting, spreading, deposition, etc. of droplets of different sizes and speeds on the banana leaf surface under the conditions of different wettability on the adaxial side and abaxial side.

In general, hollow cone nozzles showed better deposition performance. with the increase of wind speed and/or spray pressure, spray coverage and droplet density both increased first and then decreased, which was roughly the same on both sides of banana leaves. Therefore, through the reasonable selection of key components of the spray equipment and the correction of operating parameters to improve the spray deposition distribution of banana orchards, and to compensate for the conventional spray deposition and distribution performance on both sides of the banana leaves, it highlights its huge potential and application value.

## 4 Conclusion

This study carried out research on nozzle types, wind speed, spray pressure in air-assisted spraying on the spray coverage, and droplet density of the banana leaf surface. By optimizing the combination of the key components and application parameters of air-assisted spray, the wetting mechanism between banana leaves and pesticides, the main influencing factors and interaction mechanism of the droplet deposition distribution were studied, and the variation rule of droplet deposition distribution performances with key components and application parameters of air-assisted spray in the process of air-assisted spray was clarified. The main results of this research were as follows:

The three pesticides showed Janus wettability on the banana leaf surface, but the degree of their wettability was different. Both sides of the banana leaf had high adhesion, and the adaxial side had better wettability than the abaxial side. This is mainly due to the inherent surface tension between the pesticides droplets and the banana leaves itself, the lipophilic ions in the solute and the surface microstructure of the banana leaves.

Nozzle type has the greatest influence on spray coverage and droplet density, followed by wind speed, and then spray pressure. The results of 36 groups of experiments with 3 types of nozzles and 4 wind speeds show that the hollow cone nozzle AA2-3W has the best performance, the solid cone nozzle BB2.8W is the second, and the flat fan nozzle CC1105 is the worst. In addition, the performance of spraying pesticides deposited on the abaxial side of banana leaves was better than that on the adaxial side. Generally, it is recommended to use the hollow cone nozzle AA2-3W, spray pressure at 0.5Mpa, and wind speed at 3-5m/s. Under the conditions of this operating parameter, the spray coverage and droplet density of the air-assisted spray on both sides of the banana leaf was the best.

The deposition distribution performance on the abaxial side of the banana leaves was better than the adaxial side, which was mainly determined by the fixing method of the target leaf during the experiment. Under low spray pressure and/or wind speed and high spray pressure and/or wind speed, both the spray coverage and droplet density are at a relatively low level. The former is the result of insufficient and incomplete spray deposition and sparse droplet deposition, and the latter is due to the phenomenon of high droplet impact speed and/or excessive wind load making the banana leaves curl and deform, forming a stable airfoil, and causing a large number of droplets to aggregation and slippage. Therefore, it is particularly important to clarify the selection of key components and application parameters under the optimal spray deposition performance of banana leaves through experimental research. The results of this study have important guidance and reference significance for the design of air-assisted sprayers and the adjustment of operating parameters in the banana orchard.

## Data availability statement

The original contributions presented in the study are included in the article/Supplementary Material. Further inquiries can be directed to the corresponding authors.

## Author contributions

YJ conceived and designed the experiments and wrote the paper. YJ, XX, and DS performed the experiments. YJ, TJ, and BX collected data and analyzed experimental results. ZY and JD project administration, resources, funding acquisition, supervised and revised the manuscript. All authors contributed to the article and approved the submitted version.
